# Massively augmented hippocampal dentate granule cell activation accompanies epilepsy development

**DOI:** 10.1038/srep42090

**Published:** 2017-02-20

**Authors:** Christopher G. Dengler, Cuiyong Yue, Hajime Takano, Douglas A. Coulter

**Affiliations:** 1 Departments of Neuroscience, University of Pennsylvania Perelman School of Medicine, Philadelphia, PA, 19104, USA; 2 The Research Institute of the Children’s Hospital of Philadelphia, Philadelphia, PA, 19104, USA; 3 Departments of Neurology, University of Pennsylvania Perelman School of Medicine, Philadelphia, PA, 19104, USA; 4 Department of Pediatrics, University of Pennsylvania Perelman School of Medicine, Philadelphia, PA, 19104, USA.

## Abstract

In a mouse model of temporal lobe epilepsy, multicellular calcium imaging revealed that disease emergence was accompanied by massive amplification in the normally sparse, afferent stimulation-induced activation of hippocampal dentate granule cells. Patch recordings demonstrated reductions in local inhibitory function within the dentate gyrus at time points where sparse activation was compromised. Mimicking changes in inhibitory synaptic function and transmembrane chloride regulation was sufficient to elicit the dentate gyrus circuit collapse evident during epilepsy development. Pharmacological blockade of outward chloride transport had no effect during epilepsy development, and significantly increased granule cell activation in both control and chronically epileptic animals. This apparent occlusion effect implicates reduction in chloride extrusion as a mechanism contributing to granule cell hyperactivation specifically during early epilepsy development. Glutamine plays a significant role in local synthesis of GABA in synapses. In epileptic mice, sparse granule cell activation could be restored by glutamine application, implicating compromised GABA synthesis. Glutamine had no effect on granule cell activation earlier, during epilepsy development. We conclude that compromised feedforward inhibition within the local circuit generates the massive dentate gyrus circuit hyperactivation evident in animals during and following epilepsy development. However, the mechanisms underlying this disinhibition diverge significantly as epilepsy progresses.

Situated as the initial component of the canonical trisynaptic circuit, the dentate gyrus (DG) is a critical entry point to the hippocampus, regulating access of cortical input to the limbic system. Essential to this role are the sparse, selective activation properties of the DG’s principal cells, dentate granule cells (DGCs). *In vivo* recording studies have demonstrated that these neurons exhibit spatially selective firing in extremely small populations^1^,^2^,^3^. Moreover, studies examining immediate-early-gene expression, a molecular readout for sustained neuronal activity, have described exceptionally sparse labeling in DGCs, even after exposure to multiple environments or spatial cognitive tasks^4^,^5^.

This characteristic, sparse activation of DGCs is thought to enable them to participate in the execution of cognitive functions such as pattern separation and novelty detection^1^,^6^. The propensity for DGCs to generate action potentials (APs) is tightly constrained by cell-intrinsic biophysical properties^7^, ^8^, ^9^ and robust local inhibitory control^10^, ^11^, ^12^, culminating in a population of neurons that are extremely reluctant to activate^13^. However, the circuit mechanisms defining how the few responsive DGCs are recruited during cognitive tasks remain unknown.

A secondary consequence of the DG’s low excitability is its ability to restrict the relay of pathological, synchronous cortical activity into downstream hippocampal and limbic structures, regulating seizure activity in diseases such as epilepsy - a phenomenon termed ‘dentate gating’^13^,^14^. Artificially inducing failure of normal DG gating is sufficient to induce seizure generation and propagation^15^. DG circuit properties are significantly disrupted in humans with epilepsy and in animal models of the disorder. Alterations include sprouting of recurrent mossy fiber synapses^16^, molecular and cellular alterations of local inhibitory circuits^17^,^18^,^19^,^20^,^21^, aberrant neurogenesis^22^, astrocytic gliosis^23^, and alterations in the intrinsic properties of DGCs^24^. This aggregation of cellular and circuit changes in the DG accompanying epilepsy development has generated a prevalent hypothesis that its normal gating function is compromised, and this, in turn, may contribute to increased seizure propensity. However, to date, DG gate failure has primarily been described using field potential recordings, which are not cell-specific and do not identify cellular mechanisms underlying this epilepsy-associated circuit collapse.

In the present study, we used multicellular calcium imaging (MCI) to investigate possible alterations in DG circuit activation properties during and following epilepsy development in a mouse-pilocarpine model of temporal lobe epilepsy. We report that epilepsy development was accompanied by massive augmentation in the normally sparse activation of DGCs. Whole-cell patch recordings demonstrated reductions in local DG inhibitory function at time points when sparse activation was compromised. Pharmacologically mimicking changes in inhibitory receptor function in control slices was sufficient to reproduce the DG circuit collapse evident in epileptic animals. Chloride extrusion blockade elevated DGC activation in control and epileptic animals, but these effects were occluded early during the epilepsy development, suggesting altered transmembrane chloride gradients may contribute to hyperexcitability specifically at this time point. Finally, metabolic reversal of astrogliosis-mediated disinhibition in slices from chronically epileptic animals restored normal DGC activation. We conclude that the massive circuit hyperactivation evident in animals during and following epilepsy development is generated in large part by compromised local circuit inhibition, but the underlying contributory mechanisms mediating this disinhibition differ at these varying time points.

## Results

To assess changes in aggregate DG network activation during epileptogenesis, we assayed DGC proportional activation in response to perforant path (PP) stimulation using MCI in slices from control animals, and at several time points following status epilepticus (SE). MCI exploits the finding that the strong depolarization during APs induces activation of high-threshold Ca^2+^ channels. Because of this, a Ca^2+^ influx into neuronal somata occurs only during spiking, and not in response to subthreshold depolarizations. Bulk-loading cell populations with Ca^2+^-sensitive indicators transduces these AP-induced Ca^2+^ influxes into fluorescence changes and allows dynamic imaging-based assessment of APs in large neuronal populations with single-cell resolution^25^,^26^,^27^. We previously have shown that Oregon Green BAPTA-1 (OGB) used in this study is capable of detecting single APs in hippocampal neurons^26^,^27^. Use of a swept field confocal microscope capable of frame rates up to 400 Hz enhances the temporal resolution of these MCI recordings^26^,^27^.

**Figure 1:**
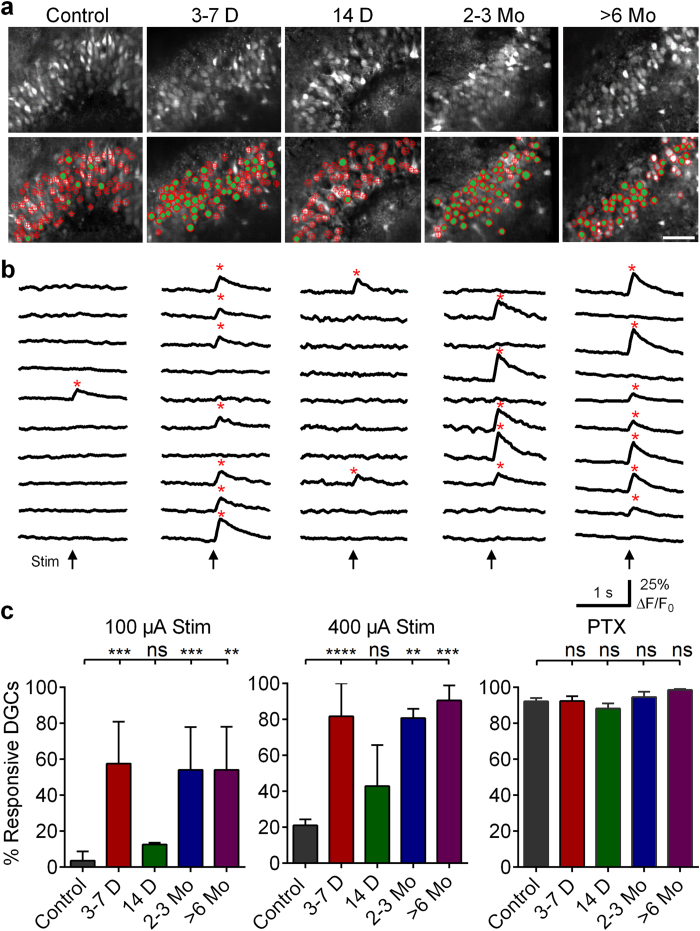
Changes in DGC activation during epileptogenesis. (**a**) Confocal micrographs of OGB-1-loaded DGCs from control mice and at defined time-points post-SE. Below are same images with overlaid ROIs depicting non-activating (hollow red ROIs) and activating DGCs (red ROIs filled green) in response to 100 μA PP stimulation. Scale bars: 50 μm. (**b**) Representative traces of time-resolved calcium imaging responses from representative ROIs selected from images above. Arrows below traces indicate the time of stimulation. * (red) denote cellular activation detected with Ca^2+^ transient detection software. (**c**) Plots of the proportional DGC activation (%) to 100 μA (left) and 400 μA (middle) PP stimulation, as well as 400 μA stimulation in the presence of 50 μM PTX (right). Note the significantly augmented DGC activation at 3–7 days, 2–3 months and >6 months post-SE compared to Controls. Samples sizes as (n [slices], replicates [mice], total number cells [ROIs]): Control: (23,9,1020); 3–7 Days: (9,6,517); 14 days: (7,3,405); 2–3 Months: (8,3,443); 6 Months: (6,3,372). Kruskal-Wallis test with Dunn’s multiple-comparison *post hoc* testing, left and center are log(value + 1) transformed to equalize variance before statistical testing, 100 μA: H(4,48) = 32.74; 400 μA: H(4,48) = 35.49, PTX H(4,48) = 6.418. Levels of significance indicated as: ns p > 0.05, *p < 0.05, **p < 0.01, ***p < 0.001, ****p < 0.0001. Histograms indicate median ± interquartile range. Also, see Supplementary Videos S1,S2,S3,S4,S5 for visualization of differences between DGC activation at each defined time point.

In response to moderate and strong PP stimulation (100 and 400 μA, ~60% and 100% maximal fEPSP [Supplementary Fig. S1]), slices prepared from control animals (control slices) displayed extremely sparse DGC proportional activation, typical of activity observed *in vivo* (Fig. 1a–c), with only 4% and 21% of DGCs responding, respectively. Subconvulsive pilocarpine treatment and/or electrode implantation had no effect on DGC activation (Supplementary Fig. S2). Sparse activation deteriorated significantly following status epilepticus (SE; Kruskal-Wallis test, p < 0.0001). At 3–7 days post-SE, activation was significantly higher, with 57% and 82% of DGCs responding (p = 0.0006 and p < 0.0001, respectively), indicating an immediate, massive collapse in the normally sparse activation of DGCs. By 14 days post-SE, a time point when ~80% of mice have become epileptic (Supplementary Fig. S3), proportional activation recovered to levels indistinguishable from controls, with only 13% and 43% of DGCs responding (p > 0.9999 and p = 0.9082). Subsequently, DGCs exhibited a secondary, permanent deterioration in sparse activation evident at 2–3 and 6 months post-SE, with 54% and 81% (p = 0.0001 and 0.0023) and 54% and 91% (p = 0.0034 and 0.0003) of cells responding to 100 and 400 μA stimulation, respectively (Fig. 1a–c). By the time this secondary collapse occurred, all animals had developed epilepsy (Supplementary Fig. S3).

To ensure all DGCs in our analysis were capable of activating, and that there were no variations in slice viability across different experimental groups following SE, we applied a saturating concentration of the GABA_A_ antagonist, picrotoxin (PTX, 50 μM), at the end of each experiment and assessed DGC activation in response to a 400 μA tetanic stimulus (10 pulses, 60 Hz). We limited analysis to viable neurons, defined as neurons that exhibited stimulus-evoked Ca^2+^ transients under these conditions. Inactive cells were assumed to be either deafferented or not viable. Inhibitory blockade combined with strong stimulation resulted in activation of nearly all DGCs (>90%) in each group (Fig. 1c), indicating that the vast majority of DGCs in our preparations were viable, had not been deafferented, and were capable of firing APs. There were no significant differences in DGC activation between groups in the presence of PTX (Kruskal-Wallis test, p = 0.17), indicating consistent levels of slice viability.

Taken together, these data demonstrate a novel, trimodal 10–20 fold elevation in DGC excitability during epilepsy development and expression. DGCs transition from a sparsely activating network in controls to one that collapses rapidly following SE. Normal activation recovers transiently, and then exhibits a secondary - likely permanent - collapse towards hyperexcitability as epilepsy manifests.

### Alterations in amplitudes of evoked Ca^2+^ transients during epileptogenesis

In addition to changes observed in proportional DGC activation, we also noted significant alterations in stimulus-elicited Ca^2+^ transient amplitudes (normalized as % ∆F/F_0_) during epileptogenesis (Fig. 1b). Neuronal Ca^2+^ transient amplitudes correlate closely with the number of APs fired by a cell^26^,^27^. These changes may capture alterations in the spike firing patterns in DGCs post-SE.

**Figure 2:**
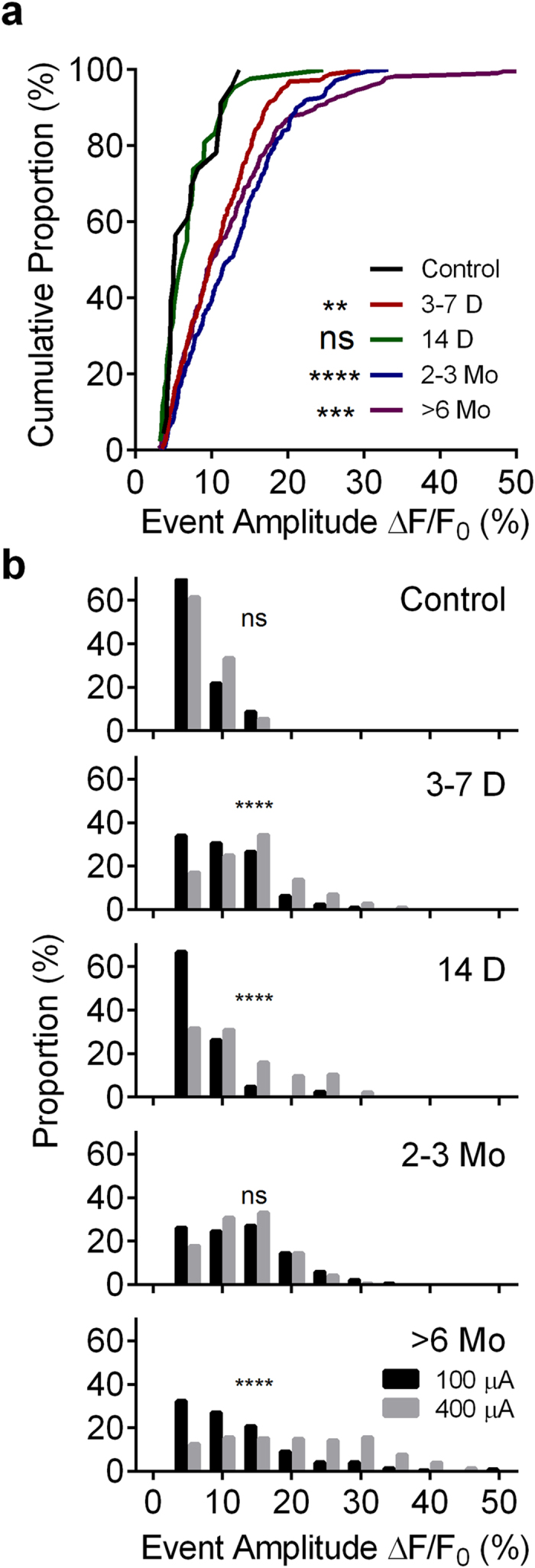
Alterations in evoked Ca^2+^ transient amplitudes during epileptogenesis. (**a**) Cumulative proportion distribution of 100 μA stimulus-evoked Ca^2+^ transient amplitudes (∆F/F_0_) during epileptogenesis (Kruskal-Wallis test with Dunn’s multiple-comparison *post hoc* testing against controls, H(4,27) = 56.5, p < 0.0001). Note the significantly increased Ca^2+^ transient amplitudes at time points corresponding to augmented DGC activation (3–7 days, 2–3 months and >6 months). (**b**) Histograms comparing distributions of 100 and 400 μA stimulus evoked Ca^2+^ transients at each time point during epileptogenesis. Kolmogorov-Smirnov test, 100 vs. 400 μA, Control: D(32) = 0.2319; 3–7 Days: D(605) = 0.2542; 14 Days; D(186) = 0.4093, 2–3 Months: D(534) = 0.052; >6 Months: D (552) = 0.3974. Sample sizes for (**a**,**b**) as (n = number OGB transients for 100/400 μA stimuli, slices, mice): Control (23/111, 11, 5); 3–7 Days (260/347,6,3); 14 days (42/146,7,3); 2–3 Months (242/294,8,3); >6 Months (223/331,6,3). Levels of significance indicated as: ns p > 0.05, *p < 0.05, **p < 0.01, ***p < 0.001, ****p < 0.0001.

In response to 100 μA stimulation, DGCs in control slices had a median Ca^2+^ transient amplitude of 5.2% ∆F/F_0_ (Fig. 2a). Responses of this magnitude are the smallest seen in this study, and likely indicate firing of single APs. In order to assess this, we conducted juxtacellular patch recordings in active, control DGCs, determined that they were firing single APs, and found the mean Ca^2+^ transient amplitude in these neurons to be 6.6 ± 0.5% ∆F/F_0_ (n = 13 cells, data not shown). This was consistent with single AP firing in the control population MCI studies. The amplitudes of cellular responses were altered significantly following SE (Kruskal-Wallis test, p < 0.0001). At 3–7 days post-SE, Ca^2+^ transient amplitudes were significantly larger than control responses (median = 9.8% ∆F/F_0,_ p = 0.0019), with the entire distribution shifted towards larger events (Fig. 2a). Lower transient amplitudes were restored 14 days post-SE (median = 6.43% ∆F/F_0,_ p = >0.9999). However, at 2–3 and >6 months post-SE, transient amplitudes were increased significantly again compared to control levels (2–3 months median = 12.3% ∆F/F_0_, p < 0.0001; >6 months median = 10.1%, p = 0.0002). Combined juxtacellular patch recordings and MCI in 2–3 month post-SE DGCs determined that single AP Ca^2+^ transients in DGCs from epileptic animals had amplitudes of 10.93 ± 1.23% ∆F/F0 (significantly larger than controls, unpaired, Welch-corrected t-test, t(14,60) = 3.253, p = 0.0055). One possible alternate contributor to larger Ca^2+^ transient amplitudes post-SE may be altered Ca^2+^ buffering in DGCs. SE down-regulates expression of calbindin, an endogenous Ca^2+^ buffering protein present in DGCs^28^ that strongly reduces global Ca^2+^ signals in neurons^29^. Reduced DGC-calbindin expression may potentially contribute to the increased signal amplitudes observed during epileptogenesis. The combined juxtacellular/imaging data described above suggests that this might be the case, as opposed to the alternative explanation of DGCs firing more APs in response to afferent stimulation.

For each group, we also measured Ca^2+^ transient amplitudes following supramaximal stimulation (400 μA, Fig. 2b) compared to moderate stimulation. Stronger stimulation failed to elicit significantly larger Ca^2+^ signals in control slices (medians for 100/400 μA: 5.2/6.6% ∆F/F_0_, p = 0.2575, significance set at p = 0.001 *a priori*), demonstrating the DG’s ability to restrict further activation of DGCs. However, supramaximal stimulation was sufficient to elicit significantly larger Ca^2+^ transients 3–7 days post-SE, (medians for 100/400 μA: 9.8/13.6% ∆F/F_0_, p < 0.0001), suggesting that the stronger stimulus generated more APs from responsive DGCs, or alternatively, that APs were broadened with supramaximal stimulation. This effect was also evident 14 days post-SE (medians for 100/400 μA: 6.4%/10.4% ∆F/F_0_, p < 0.0001).

Interestingly, there was no significant difference in Ca^2+^ transient amplitudes elicited by 100 and 400 μA stimulation 2–3 months post-SE (medians for 100/400 μA: 12.33/12.68% ∆F/F_0_, p = 0.0520). Finally, at >6 months post-SE, stronger stimulation produced a significant shift in response amplitudes to values much higher than any other groups in our study (Medians for 100/400 μA: 10.2/19.7% ∆F/F_0_, p = 0.0001). Responses of this magnitude may reflect network burst activity within the epileptic DG. Such activity may be generated in slices prepared from chronically epileptic animals as a result of the well-established recurrent mossy fiber sprouting present in models of epilepsy.

### Location of responsive DGCs within the granule cell layer

**Figure 3:**
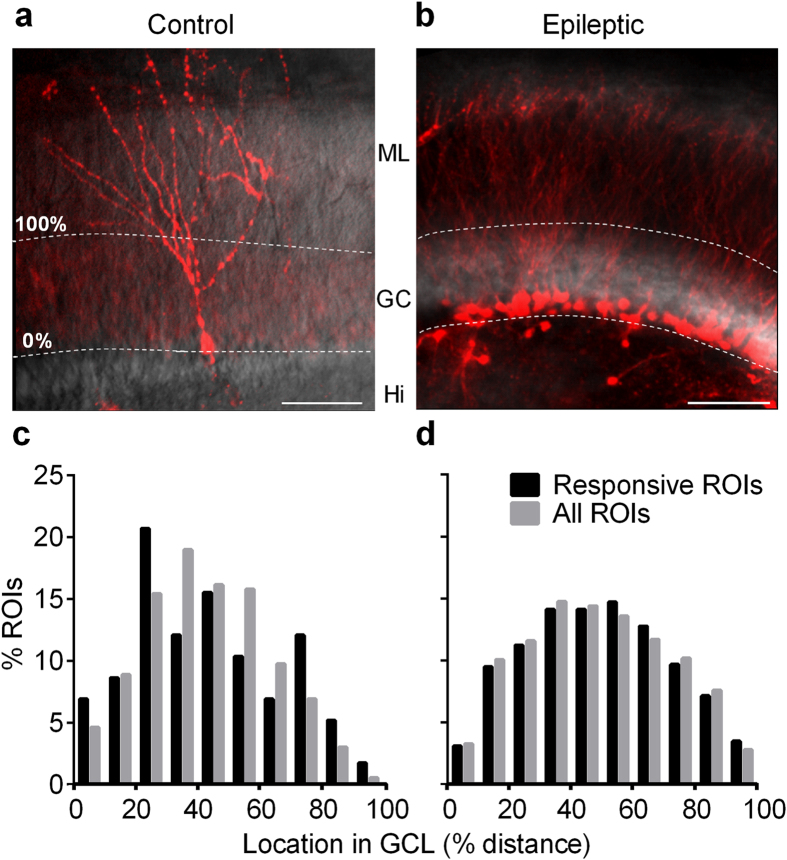
Location of responsive DGCs within the GCL. Confocal micrograph of birthdated, TdTomato-expressing, adult-born DGCs in Gli1-Cre^ERT^ x Rosa-TdTomato crossed mice. (**a**) Adult-born DGC labeled 7 weeks following 1 week of tamoxifen administration (7 d, 1/d, 150 mg/kg, I.P.) in naive animal. Scale bar: 100 microns. (**b**) Adult-born DGCs labeled 7 weeks after 1 week of tamoxifen administration (7 d, 1/d, 150 mg/kg, I.P.) immediately following pilocarpine-induced status epilepticus. Note, that in both control and epileptic slices, newborn DGCs are restricted to the inner portion of the molecular layer. White dashed lines indicate the boundaries of the granule cell layer with molecular layer (ML), granule cell layer (GC) and Hilus (Hi) labeled in center. (**c**) Histogram of responsive DGC locations and all DGC (responsive and non-responsive) locations within GCL in control slices; there was no significant difference between location distributions (Kolmogorov-Smirnov test, D (620) = 0.1132, p = 0.3281). Number of cells (Responsive/Total): n = 83/589 cells from 5 mice. (**d**) Histogram of cell locations from epileptic slices; there was no significant difference between location distributions (Kolmogorov-Smirnov test, D (1257) = 0.02967, p = 0.9393) n = 338/855 cells from 6 mice.

The DG is a unique brain structure in that it continuously generates new, functional neurons throughout life^30^. Adult-born DGCs have temporally evolving electrophysiological properties distinct from their mature counterparts. Some studies suggest that these newborn cells may be activated preferentially compared to mature DGCs^5^,^31^. New DGCs are born in the DG’s sub-granular proliferative zone, and even after maturing and establishing connectivity within the DG, typically remain confined to the inner portion of the granule cell layer (GCL). We validated this preferential localization using a transgenic approach. Figure 3a is a representative confocal micrograph of a 7 week old TdTomato-expressing adult-born DGC in a slice prepared from a Gli1-Cre^ERT^ x Rosa-TdTomato mouse, which “birthdates” DGCs with the fluorescent marker TdTomato in a tamoxifen-inducible Cre-recombinase system^32^. This cell is located near the inner GCL/hilar border; its location was stereotypic. We analyzed 13 imaging fields in slices prepared from control mice 7 weeks following tamoxifen-TdTomato birth-dating of DGCs, and found that 33/34 newborn cells were localized to the inner half of the GCL. Pilocarpine-induced SE greatly increased DGC neurogenesis (Fig. 3b). We analyzed 4 imaging fields from slices prepared from post-SE mice 7 weeks after tamoxifen-induced TdTomato DGC birthdating, and found that 327/373 of these newborn cells were localized to the inner half of the GCL. Since we used MCI to study granule cell activation exclusively in normotopic neurons within the granule cell layer, we restricted our analysis of activation position to the granule cell layer—the only region in which we had activation data. The location of Td-Tomato labeled cells within the GCL was similar in control and epileptic animals (Fisher’s exact test, p = 0.7099). Although this was our initial intent, we were unable to assess directly responses of Gli1-Cre^ERT^ birth-dated DGCs using MCI post-SE. Gli1-Cre^ERT^xRosa-TdTomato mice did not survive pilocarpine-induced SE in sufficient numbers to be useful in our MCI studies, possibly due to some unknown effect of transgene insertion.

To assess the possible contribution of newborn DGCs to overall responses in an indirect manner, we examined the location of active cells within the granule cell layer, and compared this to the total number of DGCs, generating a proportional activity/cell location plot. Figure 3c depicts the distribution of locations (plotted as % distance from the inner to outer GCL border) corresponding to DGCs activated in response to a 100 μA stimulus, as well as the location of all measured regions of interest (ROIs). There was no significant difference between these two distributions in slices prepared from control animals (active and total medians, 41%, and 41%, respectively, p = 0.3281), suggesting that newborn DGCs within the GCL do not activate disproportionally in comparison to the rest of the DGC population. Similarly, in slices prepared from epileptic mice >2 months post-SE, the distribution of responsive DGCs did not differ significantly from the entire population of imaged cells (active and total medians 49%/47%, respectively, p = 0.9393), again suggesting that the activity of GCL DGCs generated after SE also did not contribute disproportionately to overall cellular activation (Fig. 3d).

### Juxtacellular recordings of DGCs during epileptogenesis

The identity and proportion of cells that fire APs when a circuit is activated are critical aspects of appropriate neural circuit function, regulating information processing. Precise activation latency time-locked to an afferent stimulus also retains specific information about the temporal properties of the stimulus and is a critical component of information coding in a circuit. Both cell-intrinsic integrative properties of DGCs^7^,^33^ and activation of extensive feedforward and feedback inhibitory circuits^12^,^34^ determine DGC activation latencies. As such, possible alteration in DGC activation latency during epileptogenesis may reflect degraded information processing within the circuit, and may provide insight into network determinants of DG dysfunction associated with epilepsy development.

**Figure 4:**
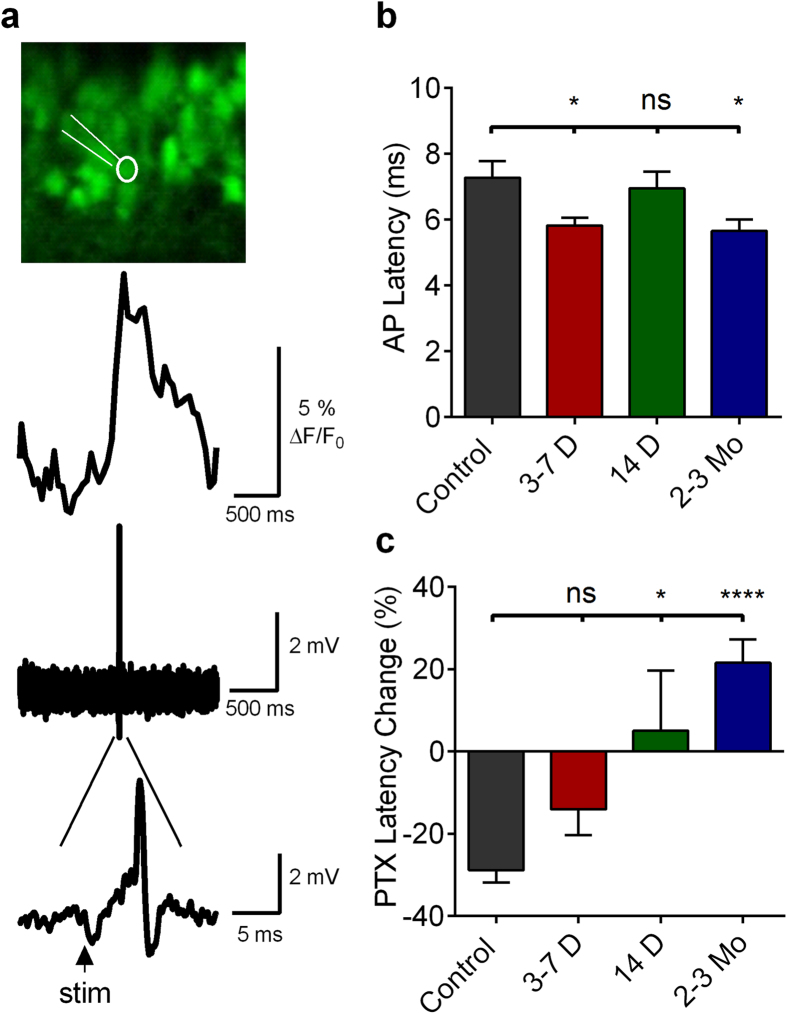
Juxtacellular recordings from active DGCs during epileptogenesis. (**a**) Top, confocal micrograph of DGCs loaded with OGB-1 in a slice prepared from a control mouse. Highlighted white is a fluorescently labeled micropipette (Alexa-488 Conjugated BSA) juxtacellularly attached to an activating DGC in a slice prepared from a control mouse. Middle, Ca^2+^ imaging transient elicited by 400 μA PP stimulus. Bottom, juxtacellular-patch recording in which individual APs can be resolved, with expanded timescale presented below depicting the latency between stimulus and AP onset can be resolved. (**b**) AP latencies obtained from juxtacellular recordings of DGCs activating in response to a 400 μA PP stimulus in slices prepared from mice at several time points post-SE. Note the significant reduction in AP latency at 3–7 days and 2–3 months post-SE compared to controls. One-way ANOVA, F(3,66) = 4.247, p = 0.0083, with Dunnett’s multiple-comparison *post hoc* testing. Levels of significance: ns p > 0.05, *p < 0.05, ****p < 0.0001 (**c**) Change in AP latencies (%) with picrotoxin application (50 μM). One-way ANOVA, F(3,50) = 9.310, p < 0.0001, with Dunnett’s multiple-comparison *post hoc* testing. Levels of significance: *p < 0.05, ****p < 0.0001) Sample sizes as (ACSF [cells], PTX [cells], replicates [mice]: Control: (16,14,5); 3–7 Days: (23,16,3); 14 Days: (15,10,3); 2–3 Months: (17,14,3). Histograms indicate means ± S.E.M. The ROUT outlier elimination method (with false discovery rate of 1%) identified 1 outlier in the 3–7 day, Control PTX, and 2 month PTX dataset; these cells were omitted from analysis.

To assess directly the temporal properties of DGC activation during epileptogenesis, we conducted MCI-guided juxtacellular patch recordings of DGCs activating in response to afferent stimulation. Since DGC activation can be extremely sparse, particularly in control slices, we employed MCI to identify activating DGCs with reliable stimulus-elicited Ca^2+^ transients, and targeted our juxtacellular patch recordings to those cells (Fig. 4a). In control slices, DGCs responding to PP stimulation activated with an AP latency of 7.28 ± 0.50 ms (Fig. 4b). Early following SE, AP latency was significantly reduced to 5.82 ± 0.23 ms (3–7 days, ANOVA p = 0.0083, with Dunnett’s multiple-comparisons test *post hoc,* p = 0.0217). Later following SE, AP latency averaged 6.94 ± 0.52 ms (14 days, not significantly different from control, p > 0.8938). Two months post-SE, AP latency decreased to 5.65 ms ± 0.35 (significantly reduced from control levels, p = 0.0149). Time points post-SE with significantly altered AP latencies also were time points in which sparse DGC firing had collapsed.

We have shown previously that both proportional activation of DGCs and the temporal windows in which APs are generated are significantly influenced by local inhibitory function^26^. To understand the role of local GABAergic inhibition in determining DGC AP latency, we applied 50 μM PTX to our preparations. As seen in Fig. 1c, PTX significantly increases proportional DGC activation, so we did not restrict our analysis to only cells activating in the absence of PTX. Inhibitory blockade significantly reduced control DGC AP latencies to 5.18 ± 0.83 ms (unpaired t-test, t(28) = 3.673, p = 0.0010), a reduction of 29% (Fig. 4c). This suggests that disynaptic feedforward interneurons may respond with sufficient rapidity to alter the onset kinetics of monosynaptic EPSPs, resulting in delay or prevention of DGC activation. This constitutes a surprising role for feed-forward inhibitory circuitry in the DG. This form of inhibition is typically thought to decrease jitter in AP activation in its targets, not to delay or prevent firing^35^,^36^.

At 3–7 days post-SE, we found that inhibitory blockade did not significantly reduce AP latencies compared to normal ACSF (ACSF mean, 5.82 ± 0.23 ms; 50 μM PTX mean, 5.00 ± 0.36 ms (unpaired t-test, t(36) = 1.974, p = 0.0561; Fig. 4b,c). That inhibitory blockade failed to affect AP latency at 3–7 days post-SE suggests either that there is diminished feedforward inhibition or that chloride reversal changes could make GABA less effective. Consistent with this latter hypothesis, blockade of chloride extrusion in control slices, using the KCC2-selective transporter blocker, R-(+)-[(2-*n*-Butyl-6,7-dichloro-2-cyclopentyl-2,3-dihydro-1-oxo-1H-inden-5-yl)oxy]acetic acid (DIOA), mimicked this AP latency decrease seen in 3–7 day post-SE animals [from 7.28 ± 0.49 ms in ACSF to 5.85 ± 0.28 ms in the presence of DIOA (unpaired, Welch-corrected t-test, p = 0.0192, control: n = 16; DIOA: n = 14, t(23.14) = 2.516), data not shown]. At 14 days post-SE, there was no difference in AP latency compared to controls. However, PTX failed to reduce AP latencies significantly when compared to control ACSF (Control ACSF mean 6.95 ± 0.51 ms, PTX mean 7.294 ± 1.01 ms, unpaired t-test, t(23) = 0.3348, p = 0.7408; Fig. 4). There was, however, a significant difference between the normalized relative effects of PTX between control mice and mice 14 days post-SE; in control slices, PTX reduced AP latencies by 28.81 ± 3.06%, while it increased AP latency 5.10 ± 14.58% in slices 14 days post-SE (one-way ANOVA, with *post hoc* Dunnett’s test, p = 0.0102; Fig. 4c). Two months following SE, PTX had a paradoxical effect: it caused a significant increase in AP latency from 5.66 ms ± 0.035 in control ASCF to 6.86 ± 0.319 ms in PTX (unpaired t-test, t(29) = 2.502, p = 0.0183; Fig. 4b,c). This PTX-induced increase in AP latency could be consistent either with excitatory actions of GABA, or with disinhibition-induced unmasking of polysynaptic excitatory inputs onto DGCs through the recurrent mossy fiber network present in epileptic animals^16^.

### Alterations in GABAergic efficacy during epileptogenesis

**Figure 5:**
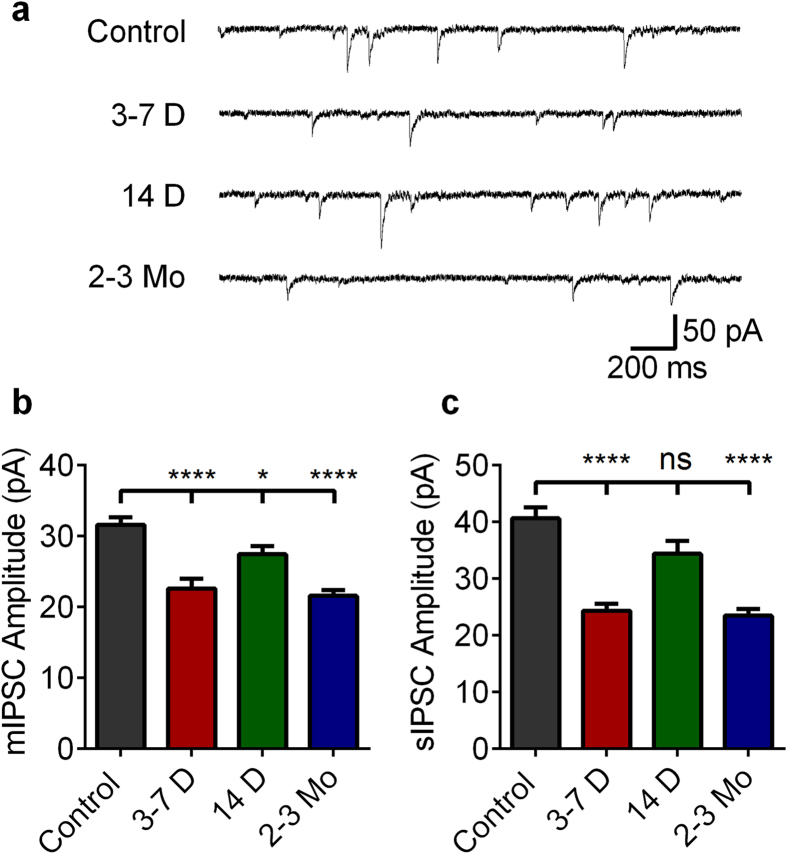
IPSC alterations during epileptogenesis. (**a**) Representative mIPSC traces of control DGCs and DGCs recorded from slices prepared from mice at 3–7 days, 14 days, and 2–3 months post-SE. (**b**) Plot of mean mIPSC amplitudes in control slices and slices prepared during epileptogenesis. (**c**) Plot of mean sIPSC amplitudes in control slices and slices prepared during epileptogenesis. (**b**,**c**) Note the significant decrease in both m- and sIPSC ampltitudes at 3–7 days and 2–3months post-SE. Cell numbers (mIPSC, sIPSC) are as follows: Control: (17, 15); 3–7 days (13, 20); 14 days: (19, 20); 2 months: (15, 17). All cells were recorded in slices prepared from at least 3 mice. One-way ANOVA with Tukey’s multiple-comparison *post hoc* testing, mIPSC F(3,60) = 17.63, sIPSC F(3,68) = 22.12, p < 0.0001. Levels of significance: ns p > 0.05, *p < 0.05, ****p < 0.0001. Histograms indicate means ± S.E.M. The ROUT outlier elimination method (with false discovery rate of 1%) identified 1 outlier in the control dataset; this cell was omitted from analysis.

GABAergic inhibition plays a predominant role in restricting DGC activation and generating the concomitant sparse activation of DGCs in control animals^12^,^37^. We, therefore, assessed inhibitory function by obtaining whole cell patch recordings of DGCs during epileptogenesis to determine whether alterations in inhibitory function accompanied changes in DGC activation properties (Fig. 5). Control mean mIPSC amplitude was 31.6 ± 1.03 pA (Fig. 5b). At 3–7 days post-SE, mIPSC amplitude decreased to 22.58 ± 1.39 pA, (a 29% reduction, p < 0.0001). mIPSC amplitude was also significantly reduced 14 days post-SE, albeit to a lesser extent, by 13% (to 27.46 ± 1.39 pA; p = 0.0170). Finally, 2–3 months post-SE, mIPSC amplitudes was again reduced by 32% relative to controls (to 21.57 ± 1.39 pA; p < 0.0001]). mIPSC frequencies did not differ between controls and post-SE groups (ANOVA, p = 0.58, Supplementary Fig. S4a).

We also recorded sIPSCs, which exhibited parallel changes to mIPSCs (Fig. 5c). sIPSC mean amplitudes were: Control: 40.66 ± 1.90; 3–7 days: 24.37 ± 1.19; 14 days: 34.42 ± 2.22; and 2–3 months: 23.48 ± 1.53 pA (mean ± SEM). sIPSC amplitudes were significantly reduced 3–7 days and 2–3 months post-SE (by 40%; p < 0.0001 and 42%; p < 0.0001, respectively). sIPSC frequencies at 3–7 days and 2–3 months post-SE did not differ significantly from controls; interestingly, sIPSC frequency was significantly increased from control levels 14 days post-SE (ANOVA with *post hoc* Dunnet’s Test, p = 0.0338, Supplementary Fig. S4b). The correlation between DGC activation and relative reduction in both m- and sIPSC amplitudes post-SE further supports compromised inhibitory function as a contributory mechanism underlying the deterioration in sparse DGC activation during both the development and expression of epilepsy.

### Disruption in inhibitory function degrades sparse DGC activation

Sparse activation of DGCs is dependent on tight inhibitory control from local GABAergic inhibitory interneurons^11^,^26^,^38^. Since a 30–40% reduction in mIPSC and sIPSC amplitude was evident during epileptogenesis (Fig. 5), we mimicked this change pharmacologically by application of the GABA_A_ receptor antagonist, PTX (5 μM) in control slices and assayed DGC activation using MCI to examine whether this level of reduced inhibitory function was sufficient to enhance DGC excitability. We selected 5 μM PTX because it reduced mIPSC amplitudes in DGCs from control slices by ~35% (Control mean 31.62 ± 1.03 pA [n = 17 cells], 5 μM PTX mean 20.64 ± 2.55 [n = 12 cells], Welch-corrected, unpaired two-tailed t-test, t[14.59] = 3.995, p = 0.0012).

**Figure 6:**
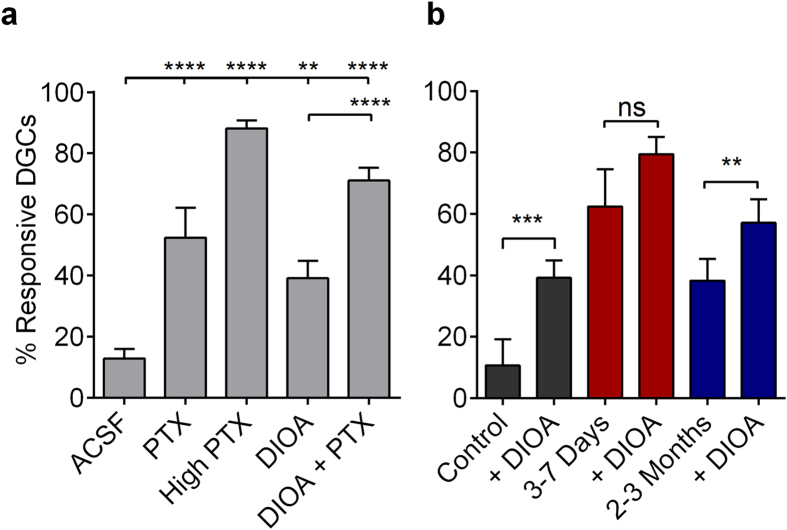
Disruption in inhibitory function degrades sparse DGC activation. (**a**) Plot of the proportional DGC activation (%) in control slices following 400 μA PP stimulation in control ACSF, 20 μM DIOA, 5 μM PTX, 20 μM DIOA + 5 μM PTX, and 50 μM PTX. Samples sizes are as follows as (n [slices], total number of cells [ROIs]), Control ACSF: (15,565); 5 μM PTX: (6, 239); 50 μM PTX: (15,565); 20 μM DIOA: (9,326); 20 μM DIOA + 5 μM PTX: (15, 565); Slices prepared from 4 naïve mice. Significance tested with one-way ANOVA, F(4.55) = 54.25, p < 0.0001, with Tukey’s multiple-comparison *post hoc* testing. (**b**) Plots of DIOA (20 μM) effects on the proportional DGC activation (%) to 400 μA PP stimulation in slices prepared from control, 3–7 days post-SE, and 2–3 months post-SE mice. Note the occlusion of DIOA effects at the 3–7 days post-SE time point. Samples sizes as (n [slices], replicates [mice], total number of cells [ROIs]), Control: (9,3,367); 3–7 days post-SE: (8,4,348); 2–3 months post-SE: (8,3,355). Significance tested with two-tailed, paired t-tests, Control: t(8) = 6.025; 3–7 days: t(7) = 1.524; and 2–3 months: t(7) = 3.956. (**a**,**b**) Histograms indicate means ± S.E.M. Levels of significance: ns p > 0.05, **p < 0.01, ***p < 0.001, ****p < 0.0001.

In normal ACSF, PP stimulation (400 μA) activated 13.0 ± 3.0% of cells (Fig. 6a). Perfusion of PTX (5 μM) significantly enhanced DGC activation ~4-fold (52 ± 9.8%; p < 0.0001), demonstrating that a reduction in inhibitory function comparable to that observed during epileptogenesis is sufficient to compromise sparse activation in DGCs. Saturating PTX concentrations (50 μM) further enhanced DGC activation to 88.1 ± 10.4% (p < 0.0001). These results implicate reduced inhibitory drive onto DGCs as a critical mechanism contributing to DGC hyperexcitability during epileptogenesis.

However, the efficacy of ionotropic GABAergic synapses depends not only on the pre- and postsynaptic function of synaptic machinery but also on the driving force of Cl^−^ ions across the synaptic membrane. Following SE, reduced expression of the Cl^−^-extruding K^+^/Cl^−^ cotransporter, KCC2, causes intracellular accumulation of Cl^−^ ions and contributes to enhanced DGC excitability via reduced inhibitory shunting efficacy of ionotropic GABA_A_ receptors^39^. This change in chloride regulation was not assessed in our whole cell patch recordings. We therefore mimicked this dysregulation in transmembrane Cl^−^ in our slice preparations by application of DIOA. DIOA perfusion (20 μM) elevated DGC activation 3-fold (from 13 ± 3.1% to 39 ± 5.6%; p = 0.0011). Combined application of 20 μM DIOA and 5 μM PTX had a synergistic effect, producing even more robust activation of DGCs (>5-fold) and a near complete collapse of dentate gating, with 71 ± 4.2% of DGCs responding under these conditions (Fig. 6a). We went on to assay DIOA (20 μM) effects on DGC activation in slices prepared from control, 3–7 days, and 2–3 month post-SE mice. DIOA enhanced DGC excitability in both control and 2–3 month post-SE, but not in 3–7 day post-SE slices (Fig. 6b). This is consistent with an occlusion effect on DIOA actions, indicating KCC2 function may be compromised at 3–7 days post-SE. These results demonstrate that compromised local GABAergic inhibitory input onto DGCs, as well as the inappropriate regulation of intracellular [Cl^−^], are each sufficient to degrade sparse activation of DGCs, and that this combined mechanism may be operative to degrade DG circuit functions 3–7 days post-SE.

### Metabolic rescue of circuit collapse in the chronically epileptic DG

**Figure 7:**
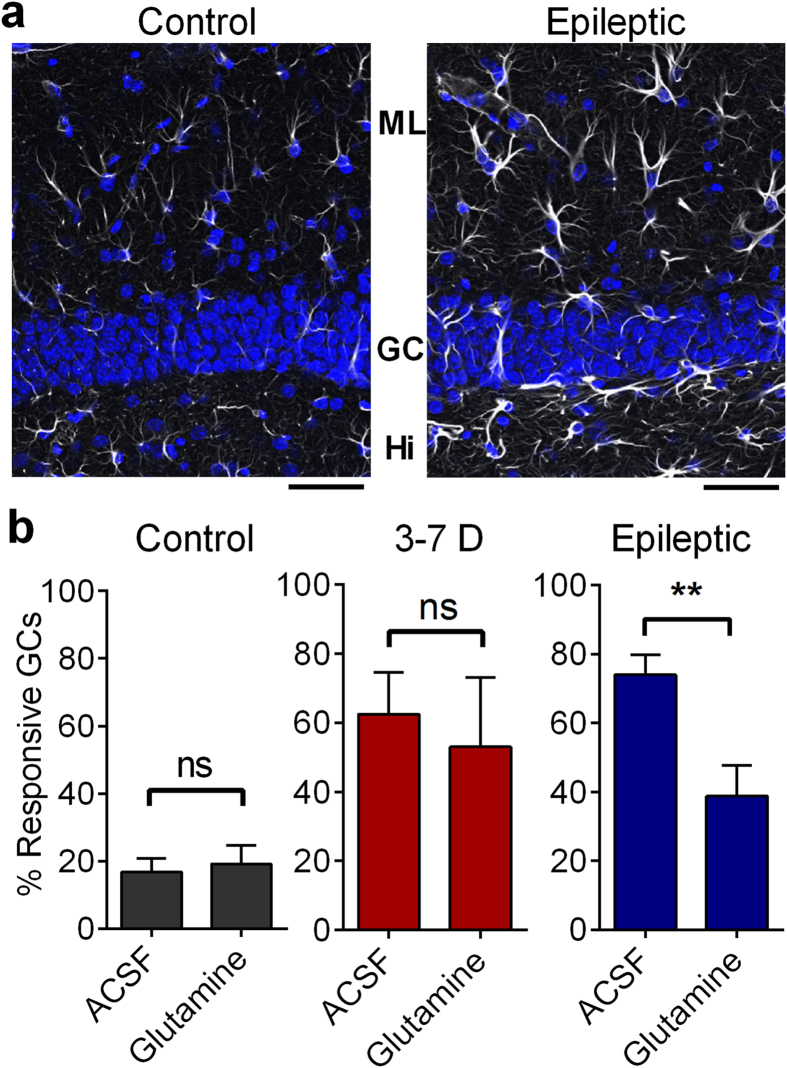
Metabolic rescue of circuit collapse in chronically epileptic DG. (**a**) GFAP immunostaining of astrocytes in control and epileptic brain slices. (Greyscale: GFAP immunostaining; Blue; DAPI labeled nuclei). Note the increased expression of GFAP and hypertrophy of astrocytes in epileptic tissue. Immunostaining repeated in 4 control and 9 epileptic slices. Scale bars represent 50 μm. (**b**) Proportional DGC activation (%) to 400 μA PP stimulation in slices from control (left), 3–7 days (middle) and epileptic mice (right, 2 months post-SE) when perfused with ACSF and 5 mM Glutamine. Notes that glutamine’s effect in reducing DGC activation was only present in epileptic mice, 2–3 months post-SE. Control: unpaired 2 sided t-test, t(15) = 0.3512, p = 0.7303; 3–7 days: Welch-corrected, two-sided, unpaired t-test, t(10.06) = 0.7106, p = 0.4935: Epilepsy: Welch-corrected, one-tailed, unpaired t-test, t(12.71) = 3.298, p = 0.0030. Right, individual data points are plotted. Sample sizes as follows, n = (ACSF slices, Glutamine slices): Control ACSF: (9, 8); 3–7 days: (8, 14); and Epileptic: (8, 8). Slices taken from at least 3 mice. Histograms indicate means ± S.E.M.

While postsynaptic disinhibition and altered chloride regulation are likely mechanisms for the collapse of sparse firing in the DG during epileptogenesis, other circuit mechanisms may contribute to the DGC hyperexcitability observed in slices from chronically epileptic mice. Reactive astrocytosis is a prominent pathology in epilepsy (and present in the DG [Fig. 7a]), and can reduce the efficacy of inhibitory neurotransmission via downregulation of glutamine synthetase^23^. In the brain, glutamine is exclusively supplied to neurons by astrocytes as the rate-limiting step in neurotransmitter recycling. Application of exogenous glutamine, which normally has no effect, can rescue this glutamine synthetase deficiency in gliotic regions of the brain by acting as the missing synthetic precursor and replenishing GABA in inhibitory presynaptic terminals^40^. In order to understand the potential role of gliosis in the epileptic DG, we treated slices prepared from control, 3–7 days post-SE, and chronically epileptic mice with 5 mM glutamine (2 hours, Fluka, Buchs, Germany), and imaged activation of DGCs via MCI. This metabolic treatment had no effects on control and 3–7 days post-SE slices, but was sufficient to partially restore normal DGC activation in slices prepared from chronically epileptic animals (Fig. 7b). In normal ACSF, 74 ± 5% of epileptic DGCs activated in response to a 400 μA PP stimulus. Glutamine treatment resulted in a significant decrease in this activation to 38 ± 9% (p = 0.0030). Glutamine treatment had no significant effect on DGC activation in control slices (p = 0.7303; Fig. 7b), or in slices prepared 3–7 days post-SE (p = 0.4935). We conclude that glutamine effects are restricted to chronically epileptic mice, and are consistent with a contribution of astrogliosis to DGC hyperexcitability in these animals. Earlier results (Fig. 4) showed that GABA blockade increased AP latency in DGCs, due to either excitatory actions of GABA or activation of recurrent mossy fibers. Here, results in 2–3 month post-SE animals demonstrate that glutamine-mediated GABA increase reduces proportional DGC activation, indicating that GABA is in fact inhibitory at this time point. It further suggests that the earlier observed PTX-effect on AP latency (Fig. 4) is likely due to the unmasking and activation of the recurrent mossy fiber network present in 2–3 month post-SE animals. These recurrent synapses are enriched in postsynaptic kainate receptors relative to normal mossy fiber terminals and PP synapses, making them more sensitive to kainate antagonists than other excitatory synapses within the DG^41^. Consistent with a role for recurrent mossy fiber synapses in proportional DG activation in epileptic animals, application of the kainate receptor antagonist (S)-1-(2-Amino-2-carboxyethyl)-3-(2-carboxy-thiophene-3-yl-methyl)-5-methylpyrimidine-2,4-dione (UBP-310, Santa Cruz Biotechnology, Santa Cruz, California)(5 μM) was sufficient to reduce DGC activation significantly in epileptic slices (from 74 ± 5% of DGCs to 54 ± 7%; one tailed, paired t-test, p = 0.0119 (Supplemental Fig. S5, right), but had no effect on control DGC activation (one-tailed, paired t-test, p = 0.7131, Supplemental Fig. S5, left).

## Discussion

This study examines DG circuit function as it transforms during and following epileptogenesis. Using MCI, we found that epilepsy development was accompanied by massive enhancement of the normally sparse activation of DGCs. Further experiments revealed alterations in DGC activation latency during epileptogenesis that were likely mediated by compromised GABAergic inhibition. Pharmacologically mimicking these changes in inhibitory synaptic function in controls was sufficient to generate the DG circuit collapse evident in epileptic animals. Circuit function in chronically epileptic mice was rescued by application of exogenous glutamine. We conclude that the massive DG circuit hyper-activation evident in animals during and following epilepsy development is generated in large part by mechanistically complex compromises in local circuit inhibition.

Although few other studies examining DG circuit behavior in epilepsy have utilized MCI techniques, as we have in the present study, many field potential and patch clamp recording studies have been published, both in post-SE rat models of epilepsy, and in hippocampal tissue resected from patients with epilepsy. There are a number of interesting similarities and differences in results obtained from rats and humans, compared to the mouse data in the present study.

The most directly comparable sets of studies to those presented here have been conducted in rats, where a number of reports have examined responses in the DG during and following epilepsy development in pilocarpine, kainate, and electrical stimulation post-SE models. A general, but variable, trend in both field potential, voltage-sensitive dye imaging, and patch clamp recording studies in post-chemically or -electrically evoked SE rats from both our own laboratory and others is that there is an early (within days post-SE) hyperexcitability in the DG^39^,^42^,^43^ accompanied by a reduction in synaptically recorded inhibition or paired pulse extrapolated inhibition in DGCs^43^,^44^,^45^. In many studies, this early DG hyperexcitability then resolves within the next few weeks^39^,^46^ but see ref. 47, accompanied by a normalization in inhibitory synaptic responses, which persists long term^44^,^45^,^48^,^49^,^50^, see^51^,^52^,^53^. The early post-SE DG hyperexcitability appears similar to the enhanced recruitment of DGCs evident in mice in the present study, as does the reduction in synaptic inhibition. However, the recovery of DG hyperexcitability and synaptic inhibition described in mice in the present study is only transient, with a later, permanent failure in both properties evident in mice (current study, see also refs 17,54). The partial recovery and then secondary collapse of appropriate DGC excitability we describe in mice may be mediated by the failed induction of a compensatory mechanism following injury, which is ineffective and suffers a secondary reversal due to ongoing seizures. In addition to species (rats vs. mice) dissimilarities potentially contributing to specific time course differences in inhibitory compromise post-SE, there are frequent divergent methodologies, including variation in the duration of SE (e.g. 40 min in the present study compared to 90 min^26^ and 120 min^17^,^21^). This could induce distinct patterns of pathology, particularly interneuron loss, which might contribute to discrepancies in m- and sIPSC frequency effects between studies^17^.

How does this compare with studies conducted in hippocampal tissue resected from patients with TLE? Although human recordings suffer from an unavoidable lack of rigorous controls, studies comparing DG excitability and inhibition in hippocampal slices have reported significant stimulus-associated hyperexcitability and disinhibition, which is most strongly apparent in tissue from patients with mesial temporal sclerosis (classic TLE), and not in tissue from patients with tumor-associated TLE, or from control rats^55^,^56^,^57^. This chronic DG hyperexcitability in epilepsy appears most similar to the profile evident in the mouse studies presented here, and not to many rat post-SE epilepsy models, which frequently exhibit little in the way of circuit alterations in normal extracellular media (discussed above).

Within the DG, sparse DGC AP firing depends on GABAergic inhibition, and the initial determination to activate is accomplished within milliseconds. This implicates feedforward inhibition as a prime candidate regulator of DGC sparse activity. Feedforward inhibition is tuned to define an integrative window in principal cells, temporally locking circuit output to afferent input, and sharpening the timing of circuit responses^35^,^36^. In the DG, this function is modified to regulate strongly the ability of DGCs to activate^12^. Experimental support for this unique role of feedforward inhibition includes the effects of GABA antagonists to increase the proportion of cells that activate in a circuit lacking recurrent excitatory collaterals (Figs 1c and 6) and to reduce activation latency (Fig. 4c). The KCC2 antagonist, DIOA, also reduces DGC activation latency, also consistent with early GABAergic inhibition regulating DGC firing. Feedforward inhibition can modulate cell firing in cortical circuits^37^,^58^, but the magnitude of this effect in the DG constitutes an uncommonly extreme example, where feedforward inhibition prevents most cells from firing, while also regulating the timing of firing in the residual active population^11^.

Active DGCs exhibited no preferential localization within the GCL (Fig. 3). The cell somata of adult-born DGCs coalesce almost exclusively in the inner portion of this layer in both control and epileptic animals (Fig. 3a,b, see also ref. 22). The lack of preferential location of active DGCs provides indirect evidence that, in our studies, both adult-born and mature DGCs within the GCL contribute equivalently to ensemble activation. This contrasts recent reports predicting enhanced excitability of adult-born DGCs during synaptic processing within the DG^11^,^31^,^37^, and during cognition^5^. However, we characterized only the activity of normotopic DGCs and did not examine ectopic DGCs within the hilus, distant from our imaging regions within the DG cell layer. Many adult-born DGCs in epilepsy migrate to ectopic locations within the hilus and integrate abnormally into hippocampal circuitry^59^. The accumulation of these ectopic, but not normotopic, adult-born DGCs correlates with epilepsy severity^60^.

The normal sparse DGC activation evident in control animals increases 10–20 fold during and following epilepsy development (3–7 d and >60 d post-SE, respectively). Based on previous studies examining hippocampal alterations during epileptogenesis and in chronically epileptic animals, the underlying mechanisms responsible for this massive alteration in circuit function are likely to differ at these two distinct phases following SE. However, in both cases, compromised GABAergic inhibition is a likely contributor, given the pivotal role of interneurons in regulating DGC activation. Immediately following SE, there is compromised regulation of transmembrane chloride levels in DGCs, resulting in elevated intracellular chloride, compromised GABAergic inhibition, and loss of the gating function of the DG^39^. In addition, patch studies conducted immediately following SE in the rat pilocarpine model of epilepsy (3–7 days) have demonstrated reductions in inhibitory function in DGCs^17^,^45^ similar to those we report in mice (Fig. 5). To evaluate the possible contributions of these two mechanisms to DGC hyperactivation post-SE, we generated similar effects in control DG through partial blockade of GABA receptors and/or perfusion with a KCC2 blocker, and found that both of these manipulations were sufficient to elicit enhanced DGC activation similar to that seen 3–7 d post-SE (Fig. 6a). In addition, the action of DIOA to increase DGC activation was occluded in slices prepared from 3–7 days post-SE but not in chronically epileptic (2–3 months post-SE) mice, further supporting the specific role of chloride disequilibrium in DG hyperexcitability during epilepsy development (Fig. 6b).

As epilepsy manifests later following SE, additional cellular and circuit processes also emerge within the hippocampus, distinct from those seen during epileptogenesis. These other factors may also have a prominent role in the DGC hyperactivation evident in epileptic animals. In addition to the disinhibition evident both 3–7 and >60 d post-SE (Fig. 5), chronically epileptic animals display marked gliosis (Fig. 7a). Astrogliosis, a hallmark of temporal lobe epilepsy, is associated with downregulated expression of glutamine synthetase, the astrocyte-specific keystone enzyme in the glutamate-glutamine cycle^23^,^40^. The astrocytic glutamate-glutamine cycle mediates neurotransmitter recycling in CNS synapses, and compromised function of this cycle rapidly depletes synaptic GABA and generates disinhibition^40^,^61^. Within the brain, glutamine is exclusively generated by astrocytes. Glutamine has no effect on control brain^40^,^62^ since this amino acid is normally not rate limiting in neurotransmitter synthesis. To determine whether astrogliosis may contribute to DGC hyperactivation in epileptic animals, we incubated slices prepared from control, early (3–7 days post-SE), and chronically (2–3 months post-SE) epileptic animals in exogenous glutamine. We found that this reversed DGC hyperactivity specifically at the 2–3 month post-SE time point. This suggests that gliosis may be a significant contributor to circuit collapse evident in chronically epileptic mice. In combination, these data demonstrate that, although the circuit disruptions evident during and following epilepsy development appear similar in extent, they are due to distinct underlying mechanisms, and so would require different therapeutic manipulations to restore normal DG function at these two time points during the disease process.

Given that the sparse ensemble activation of DGCs is a critical determinant of both hippocampal-dependent cognitive function and regulation of aberrant excitability in the limbic system, erosion in this circuit property may play a significant role both in cognitive comorbidities and seizure propensity in epilepsy. The time course of emergence and persistence of excess DGC activation links this aberrant circuit property both with the induction and expression of epilepsy. Studies providing mechanistic insight into how epilepsy development alters the basic circuit properties of hippocampal structures may be important not only in targeting new therapies for the treatment of seizures and the cognitive comorbidities accompanying epilepsy, but also, ultimately, in therapies designed to prevent and/or cure this disorder in at-risk patients.

## Methods

### Animals and tissue preparation

All animal use was performed in accordance and with the approval of the Children’s Hospital of Philadelphia’s Institutional Animal Care and Use Committee and all methods were in accordance with the relevant guidelines and regulations. Male C57BL/6 were used in this study, except for in (Fig. 3a,b), where Gli1-Cre^ERT^ x Rosa-TdTomato mice (C57BL/6 background) were used. Naïve control mice were aged 9–11 weeks, except for two older control mice aged 130–150 days in Fig. 1. Control mice were housed with up to 4 animals per cage. Mice that underwent pilocarpine-induced SE were singly housed post-SE. Vivarium light/dark cycle was 12/12 hours. For slice preparation, mice were perfused transcardially with ice cold, oxygenated (95% O_2_/5% CO_2_) artificial cerebrospinal fluid (ACSF) in which NaCl partially replaced an equal osmolarity concentration of sucrose. Sucrose ACSF was composed of (in mM): 87 NaCl, 2.5 KCl, 1.25 NaH_2_PO_4_, 75 sucrose, 10 glucose, 26 NaHCO_3_, 0.5 CaCl_2_-2H_2_O, and 4 MgSO_4_. After perfusion, brains were removed, and submerged in ice cold sucrose ACSF. Horizontal hippocampal-entorhinal cortex slices (350 μm) were cut using a Vibratome VT1200S (Leica, Wetzlar, Germany). Slices were then incubated in a calcium indicator loading chamber and humidified with 95% O_2_/5% CO_2_ at 37 °C. The calcium indicator loading solution was composed of 4 μl of 0.5% Oregon Green BAPTA-1 AM (OGB; Invitrogen), 4 μl of 20% pluronic acid (Sigma) in DMSO, 4 μl of 15% Cremophor (EL) (Sigma) in DMSO (Sigma) and 4 ml of oxygenated sucrose ACSF. Slices were loaded for 15 min and then washed and incubated in fresh oxygenated ACSF (composition in mM: 125 NaCl, 2.5 KCl, 1.25 NaH_2_PO_4_, 10 glucose, 26 NaHCO_3_, 2 CaCl_2_-2H_2_O, 1 MgSO_4_) at room temperature for at least 45 min to allow AM-ester linkages to cleave. Slices were then stored in oxygenated ACSF for up to 6 h without any evident deterioration of responses. For optical and electrical recordings, slices were placed in a 36 °C heated submersion recording chamber, instrumented with electrodes, and allowed to acclimate for 10 minutes before commencing recordings.

### Pilocarpine-induced status epilepticus (SE) model of epilepsy

Mice aged 6 weeks were injected with scopolamine (1 mg/kg), and 30 min later with pilocarpine (350 mg/kg). This triggered SE. Forty minutes after SE onset, diazepam (5 mg/kg) was administered to quell seizure activity. Ages of pilocarpine-SE mice varied depending on time post-SE as tracking circuit and cellular properties developing following SE was the primary goal of this study. Time post-SE is reported in each figure.

### Video/EEG monitoring

Seizure events were monitored using video behavioral monitoring as well as depth electrode EEG recording to confirm the manifestation of epilepsy in all mice at time points >2 months post-SE. EEG recordings were performed using a Stellate-Harmonie (Stellate Inc., Montreal, Canada) 16-bit, 32 channel digital EEG machine, sampling at 200 Hz. Cortical electrodes consisted of self-tapping screws and hippocampal electrodes of bipolar 0.005 inch stainless steel wire. The depth electrode was placed into area CA1 of the right hippocampus. Cortical electrodes were placed directly in front of the Bregma suture on both sides of the midline. Once in the correct position, all electrodes were held in a six pin pedestal. Ground and reference electrodes were placed directly behind the Lambda suture on either side of the midline. The pedestal of electrodes and screws were further held in place by dental cement. EEG tracings were reviewed to detect electrographic seizures. If a seizure was suspected, the occurrence of a behavioral seizure was confirmed. The time course and prevalence of epilepsy development in our mouse model are depicted in Supplementary Fig. S3. Epilepsy onset was rapid, with 50% of animals developing epilepsy within 5 days, and occurred in all animals by 35 days post-SE. Additionally, we confirmed that EEG electrode implantation and headcap instrumentation, with or without subconvulsive pilocarpine treatment (1/10^th^ dose pilocarpine, 35 mg/kg) did not induce epilepsy development, (Supplementary Fig. S3) nor did it alter DGC responsiveness in our MCI recordings (Supplementary Fig. S2).

### Microelectrode stimulation and recording

For stimulation of PP granule cell afferents, a 125 μm diameter concentric, bipolar tungsten stimulating electrode (FHC Inc., Bowdoin, ME) was placed ~100 μm on the entorhinal cortical side of the hippocampal fissure, in a region neighboring the suprapyramidal blade and apex of the GCL (defined as the midpoint between the suprapyramidal and infrapyramidal blades;). For extracellular recordings, a glass recording microelectrode was positioned in the middle of the DG molecular layer. Recording electrodes were placed >500 μm away from the stimulating electrode and outside the imaging field to avoid stimulus and excitation laser-indued artifacts.

### Stimulus intensity standardization

PP stimulus intensity was standardized across groups using local field EPSP slope and amplitude measurements recorded using glass microelectrodes (5–7 MΩ resistance, filled with ACSF) positioned in the middle third of the molecular layer to assess activation of the medial PP. Input-output relationships normalized to maximal response were invariant across all experimental groups (Fig. S1). In these studies, two stimulus intensities were used regularly, 100 μA and 400 μA, which corresponded to ~60% and 100% maximal stimulation. Additionally, local field potential recordings were monitored online during the optical recordings to verify the ongoing viability of slices. Local field potentials were recorded using an Axopatch 200B amplifier (Molecular Devices, Sunnyvale, CA). Electrical data were collected using Clampex 10 (Molecular Devices) software.

### Juxtacellular loose-patch recordings

Juxtacellular loose-patch current-clamp recordings were conducted in OBG-loaded slices to assess AP firing and timing associated with calcium transients in DGCs. Patch electrodes (5–7 MΩ resistance, filled with ACSF) were fluorescently labeled with 0.04% Alexafluor 488 conjugated bovine serum albumin (A13100, ThermoFischer), and then positioned onto DGCs of interest. Negative pressure was applied to acquire and maintain low resistance seals of >30 MΩ. Seal quality was monitored during recordings. Current-clamp recordings were made using a Multiclamp 700B amplifier (Molecular Devices) and sampled at 20 kHz with a Digidata 1332A analog-digital converter (Molecular Devices). Electrical data were collected and analyzed in Clampfit 10 (Molecular Devices).

### Confocal multicellular calcium imaging (MCI)

For confocal MCI, Oregon Green BAPTA-AM (OGB)-loaded slices were imaged using a Live Scan Swept Field Confocal Microscope (Nikon Instruments, Melville, NY) equipped with an Ar/Kr ion laser (Innova 70 C, or Solid State OBIS laser; Coherent, excitation wavelength 488 nm) operated with NIS-elements software (Nikon). Images were acquired using a water immersion 40X lens (NA = 0.8) and a Cascade 512 + CCD camera (Photometrics, Tuscon, AZ). This system allowed capture of images at 55 frames/s with a frame resolution of 256 × 256 pixels for these studies.

### MCI calcium transient data analysis and code availability

For MCI, the field of view at 40× magnification allowed us to capture ~30–70 OGB-loaded cells per image, and when possible, with multiple regions studied per slice. Manual construction of small oval or round regions of interest (ROI) encompassing the soma was used to analyze the activity of these cells. The average fluorescence intensities within ROIs were calculated as a function of time and exported as a text file^27^. Response to the electrical stimulus was quantified with a custom-written Matlab GUI code. Briefly, the average intensity within ROIs over time (calcium transient trace) that had been calculated in Nikon Elements software were imported as a text file in Matlab. Change in fluorescence relative to background fluorescence (ΔF/F_0_) was calculated, processed using a Savitzky-Golay smoothing filter, and derivatives of the traces were calculated for each ROI. An initial threshold value for detecting a local maximum in the derivative trace was estimated based on the entire trace for each ROI. With a sliding window, timing for a local maximum of the derivative trace was detected along with the timing for zero-crossing values for each local maximum event. The area under the curve for the event was used as the second threshold to discriminate stimulus response from noise. After determining the stimulus response, the local maximum, onset time, and 90% decay time point was estimated in ΔF/F_0_ trace for each ROI. In this algorithm, since the number of events within a trace is unlimited, it is possible to detect multiple peaks in a trace in response to a slow stimulation rate (~1 Hz) as well as spontaneous activity. The Matlab GUI code also included manual supervision/inspection steps to manually reject incorrectly identified stimulus responses. The code is available upon request. All event and related properties (ROI I.D. number, peak I.D. number, onset time, maximum ΔF/F_0_ value, peak timing, 90% decay time, a parameter for an exponential decay fit in decay portion of the trace) were recorded and exported as Excel files for aggregate analysis. Each cell’s activity was observed over time and across conditions using an ID number attached to the ROI delineating each cell. Picrotoxin (PTX, 50 μM), a GABA_A_ receptor antagonist, was applied at the end of experiments to confirm that individual neurons were able to respond to the afferent stimulus. Cells that were inactive in both control and PTX conditions were excluded from further analysis. Slices in which 70% of cellular ROIs did not activate in the presence of PTX were considered damaged or deafferented and excluded from analysis (<5% slices).

### m- and sIPSC recordings

mIPSCs were recorded in ACSF that, along with DNQX (10 μM) and D-AP-5 (50 μM), included tetrodotoxin (TTX; 1 μM) to block APs, sIPSCs were recorded without TTX. Whole-cell voltage-clamp recording of mIPSCs was conducted using a high-chloride internal pipette solution, which resulted in an inward chloride current with cells clamped at −76 mV (corrected for liquid junction potential in Clampfit). The pipette solution consisted of the following (in mM): 100 CsCH_3_O_3_S, 50 CsCl, 3 KCl, 0.2 BAPTA, 10 HEPES, 1 MgCl_2_, 2.5 Phosphcreatine-2Na, 2 Mg-ATP, and 0.25 GTP-Tris, titrated to pH 7.2–7.3 with 3 M CsOH (osmolarity 280–290 mOsm). In all experiments, lidocaine N-ethylbromide (QX-314; 5 mM) was added to the pipette solution on the day of the experiment. Synaptic currents were recorded using an Axopatch 700B amplifier (Molecular Devices), filtered at 2 kHz, sampled at 20 kHz, digitized (Digidata 1320 A; Molecular Devices), and stored for off- line analysis (using Minianalysis software written in IGOR Pro; Wavemetrics, Lake Oswego, OR) (Hwang and Copenhagen, 1999). Access resistance stability (10–18 MΩ; 80% compensation) was monitored using a 2 mV voltage step applied every 120 s, and data from cells were discarded when >15% change occurred.

### Histology and immunocytochemistry

Animals were perfused with 4% paraformaldehyde in phosphate buffered saline. Horizontal hippocampal-entorhinal cortex slices (60 μm) were cut using a vibrating tissue slicer (Vibratome VT1200S, Leica). Slices were blocked for 4 hours from non-specific binding with 5% normal goat serum in PBS with 0.4% Triton-X 100 (Sigma, St. Louis, MO) at 4°. Slices were incubated in rabbit anti-GFAP polyclonal antibody (Sigma, St. Louis, MO; Cat. #G4546; recommend for human, mouse and rat GFAP) diluted 1:400 in blocking solution for 12 hours at 4°, and washed 3x in PBS with 0.2% Triton-X-100 for 15 minutes. Slices were then incubated in Alexa Fluor 633 conjugated goat anti-rabbit highly cross-adsorbed IgG (H + L) (Thermo Fisher, Carlsbad, CA; Cat. #A21071) and DAPI (Thermo Fisher diluted 1:1000) secondary diluted 1:400 diluted in blocking solution for 1 hour at room temperature. and then washed 3× for 20 minutes. All incubations and washes occurred on a tissue rocker. Slices were mounted on glass slides in SlowFade Gold mounting media (Thermo Fisher). Images were collected using an Olympus FV1000 confocal microscope.

### Statistical analysis

N values for experiments were determined based on variance estimates derived from previous similar studies^26^,^27^,^40^,^45^. Datasets were tested for normality with a Kolmogorov-Smirnov test to determine appropriate statistical testing methods between groups. Differences in variance between groups were tested with a Brown-Forsythe test. If unequal variances were detected, data were log(value + 1) transformed to satisfy statistical testing assumptions of equal variance. For comparisons between 2 groups, significance was tested using either one- or two-way *t* tests, or for non-normally distributed data, a Mann–Whitney *U* test. For multiple group comparisons, statistical significance was tested using either a one- or two-way ANOVA, with *post hoc* Tukey’s and Dunnett’s tests for multiple-comparisons correction, or for non-normally distributed data, a Kruskal-Wallis test with *post hoc* Dunn’s test for multiple-comparisons correction. Center values from normally distributed datasets are expressed as mean ± SEM; center values from non-normally distributed datasets are expressed as median ± interquartile range. Differences between population distributions were assessed using a Kolmorgorov–Smirnov test. Contingency tables were analyzed with Fisher’s exact test. In all statistical testing, *p* values < 0.05 were considered evidence of statistical significance, unless explicitly stated otherwise. Statistical test scores and degrees of freedom (DOF) are reported as score (DOF between groups, DOF between samples), or simply, score (DOF samples) when two groups are compared.

### Blinding

Animal selection for naïve or SE groups was random. For MCI studies, experimental blinding was not entirely possible because of obvious behavioral changes in mice following SE. However, time post-SE was blinded where possible. Following collection of MCI images, data analysis was conducted blinded. For whole cell patch recordings and analysis, the experimental investigator was blinded to animal identity.

## Additional Information

**How to cite this article**: Dengler, C. *et al*. Massively augmented hippocampal dentate granule cell activation accompanies epilepsy development. *Sci. Rep.* **7**, 42090; doi: 10.1038/srep42090 (2017).

**Publisher's note:** Springer Nature remains neutral with regard to jurisdictional claims in published maps and institutional affiliations.

## Supplementary Material

Supplementary Information

Supplementary Video S1

Supplementary Video S2

Supplementary Video S3

Supplementary Video S4

Supplementary Video S5
