# Supplementary material for: Massively augmented hippocampal dentate granule cell activation accompanies epilepsy development
Source: Sci Rep. 2017 Feb 20;7:42090. doi: 10.1038/srep42090 (PMC5316990; doi:10.1038/srep42090)
Supplement: Supplementary Information [file srep42090-s1.pdf]

Massively augmented hippocampal dentate granule cell activation accompanies epilepsy development.

Christopher Dengler(1), Cuiyong Yue(4), Hajime Takano(2,4), and Douglas A. Coulter(1,3,4)

Departments of Neuroscience(1), Neurology(2), and Pediatrics(3), University of Pennsylvania

Perelman School of Medicine, Philadelphia, PA, 19104 and

The Research Institute of the Children's Hospital of Philadelphia, Philadelphia, PA, 19104 (4)

Author for Correspondence and Proofs:

Douglas A. Coulter, Ph.D.

3615 Civic Center Blvd.

Abramson Pediatric Research Center Rm 410D

Philadelphia, PA 19104-4318

email: coulterd@email.chop.edu

Phone: (215) 590-1937

Fax: (215) 590-4121

SUPPLEMENTARY INFORMATION

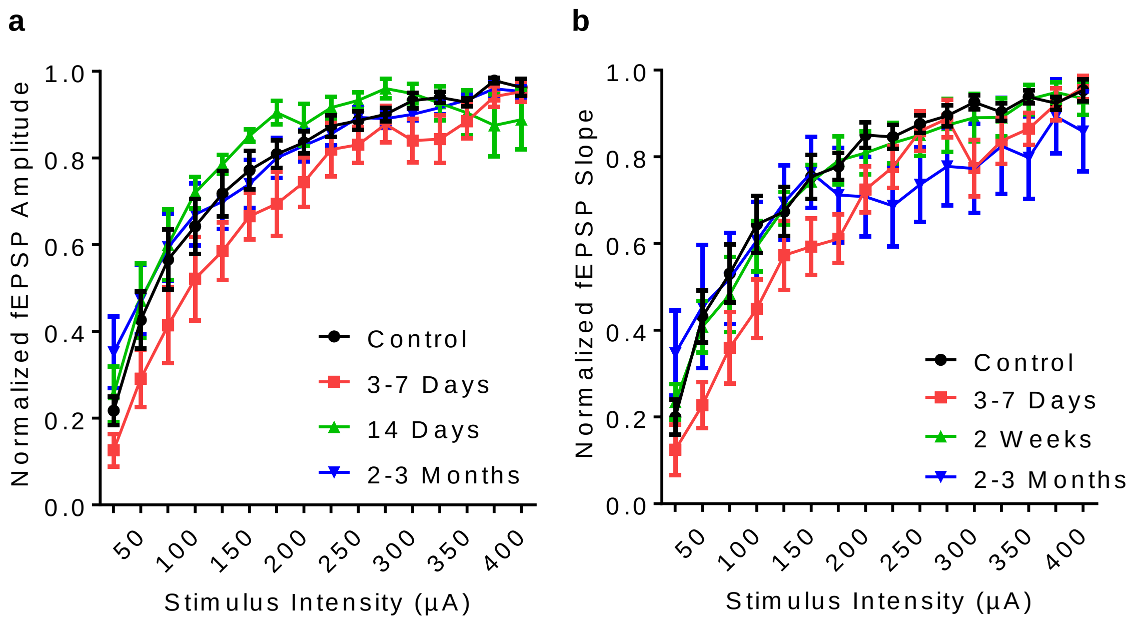

Supplementary Figure S1, Input-output relationships of perforant path stimulation during epileptogenesis. To assess the input-output relationship of stimulus intensity to DG stimulus response in DG dendrites during epileptogenesis, we conducted extracellular field recordings with our recording electrode placed in the middle third of the molecular layer and recorded responses to single 200 μs electrical stimuli applied to the angular bundle of the perforant path approximately 100 μm away from the DG molecular layer. **a**, fEPSP amplitudes normalized to maximal value plotted against stimulus intensity. Input-output relationships following pilocarpine-SE were not significantly different from controls. (2-way ANOVA, $F(45,400) = 0.7478$, $p=0.8842$). **b**, fEPSP slope normalized to maximal value plotted against stimulus intensity. Input-output relationships were not significantly different from controls following pilocarpine-SE. (2-way ANOVA, $F(45,336) = 0.6979$, $p=0.9293$). n (slices): Control n = 9; 3-7 days, n = 4; 14 days, n = 6, 2-3 months, n= 6. Slices prepared from at least 3 animals.

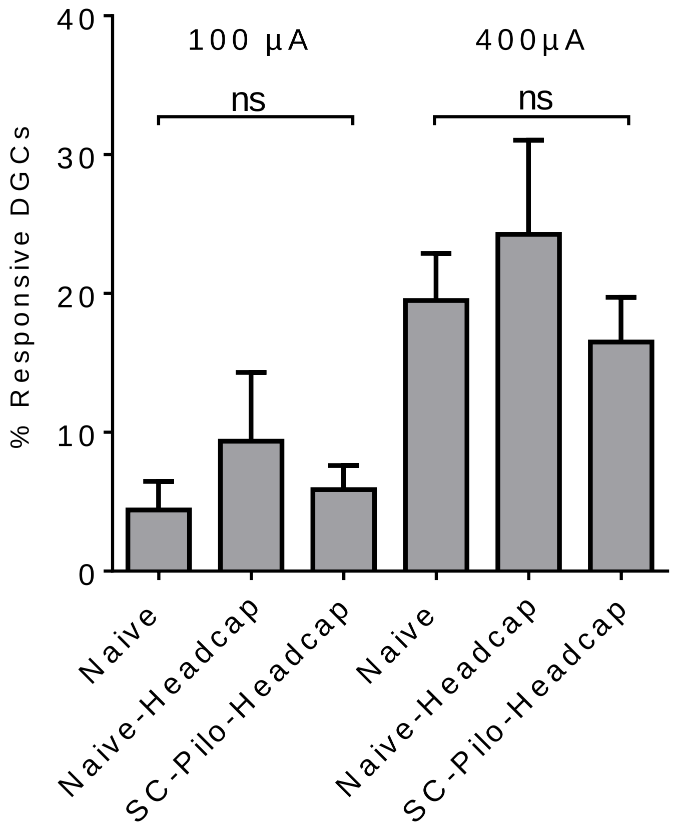

Supplementary Figure S2, EEG-instrumentation (headcaps) and sub convulsive pilocarpine treatment does not alter DGC responsiveness. Proportional activation of DGC% in slices prepared from naïve control mice, naïve mice implanted with recording headcaps, and mice subjected to subconvulsive pilocarpine (30mg/kg pilocarpine (10%) dose and 2.5 mg/kg diazepam quelling) implanted with recording headcaps, 100 µA (left) and 400 µA (right) PP stimulation. Samples sizes as (n [slices], replicates [mice], total number cells [ROIs]): Control: (11, 5, 557), Naïve-headcap: (5, 3, 251); SC-Pilo-Headcap: (7, 3, 255). ANOVA, 100 µA: F[2,20] = 0.7769, p = 0.4732 ; 400 µA: F[2,20] = 0.6704, p = 0.4732. Histograms indicate mean ± S.E.M.

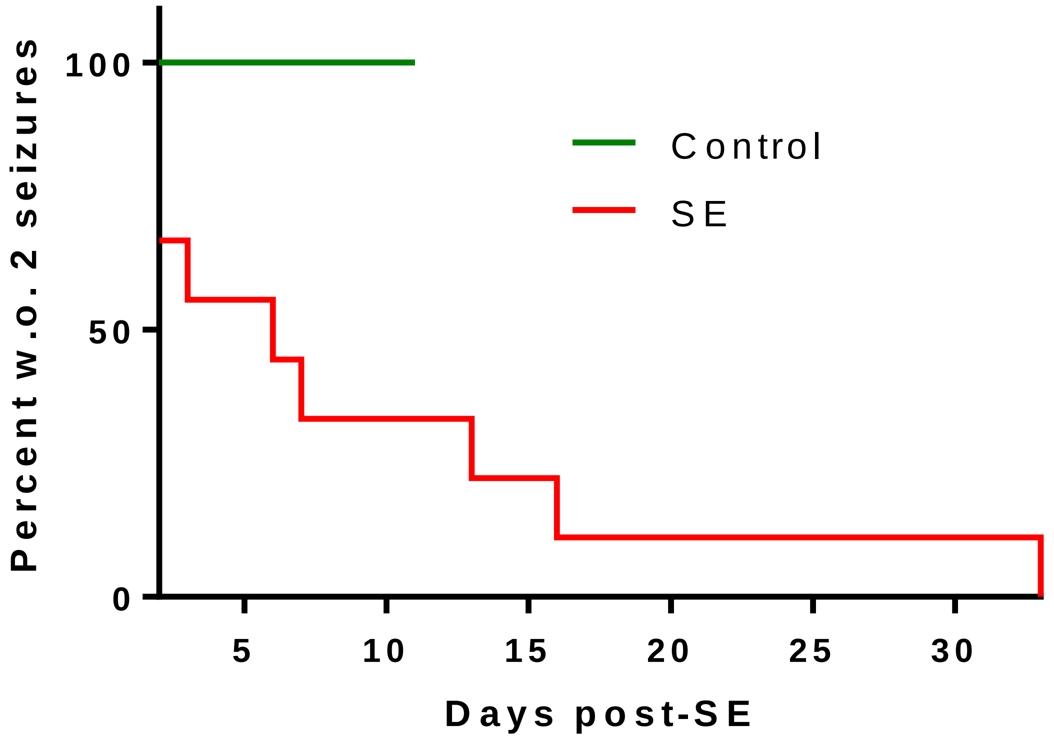

Supplementary Figure S3, Meier Kaplan survival curve depicting epilepsy onset in pilocarpine treated mice. Epilepsy onset is defined as day on which 2nd recurrent, spontaneous, seizure occurred, excluding the first 2 days following SE in which some acute post-SE seizures were noted. By 8 days post SE, 50% of SE mice were confirmed as epileptic and all mice became epileptic by day 33, n = 9 pilocarpine treated mice. Control mice (naïve headcapped, n = 3, and subconvulsive pilocarpine treated - headcapped mice, n = 3) exhibited no seizures in 11 days of recordings, total n = 6.

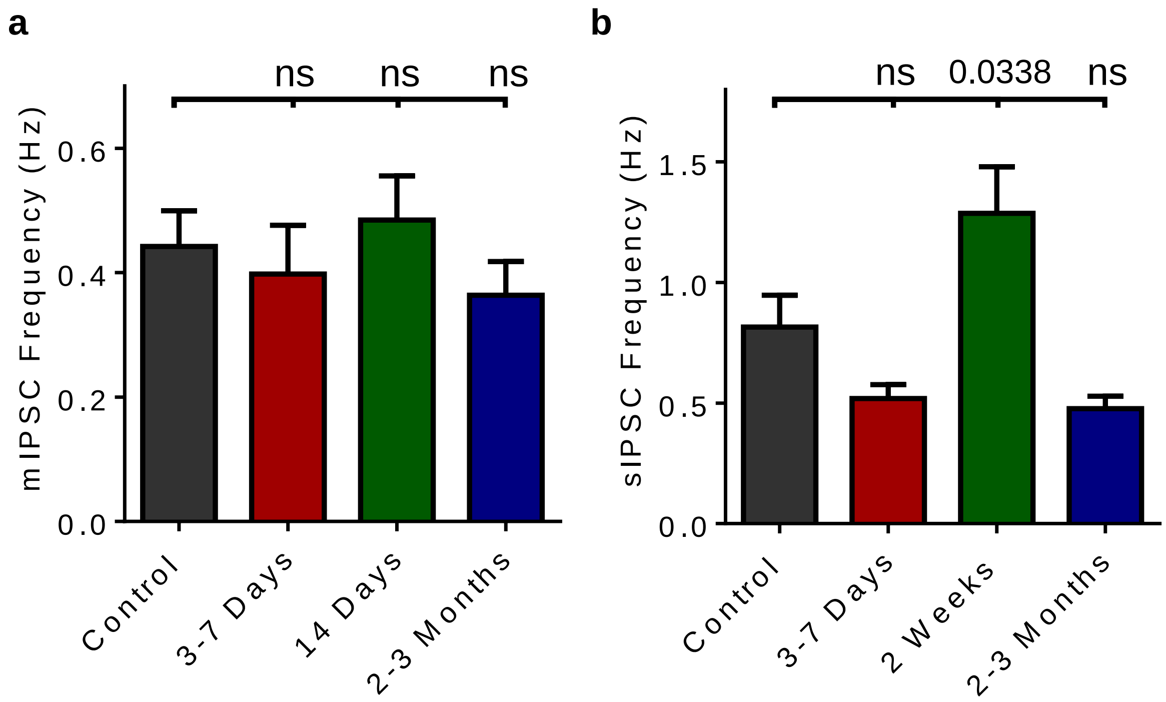

Supplementary Figure S4, m- and s-IPSCs during epileptogenesis. **a**, Plot of mIPSC frequencies in control slices and slices prepared during epileptogenesis. **b**, Plot of sIPSC frequency in control slices and slices prepared during epileptogenesis. (**a, b**) Cell numbers (mIPSC, sIPSC) are as follows: Control; n = (17,15); 3-7 days: n = (13,20); 14 days: n = (19,20), 2 months n = (15,17). All cells were recorded in slices prepared from at least 3 mice. One way ANOVA with Dunnett's multiple-comparison *post-hoc* testing, mIPSC $F(3,60) = 0.6592$, $p = 0.58$; sIPSC $F(3,68) = 9.272$, $p < 0.001$. Histograms indicate mean ± S.E.M.

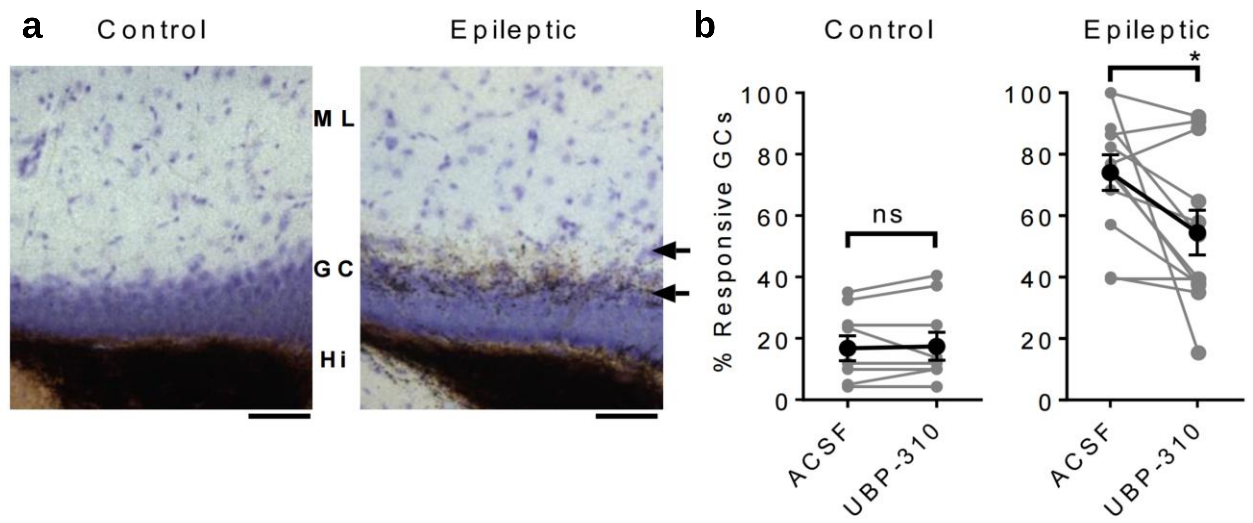

Supplementary Figure S5

a, Timm's staining of mossy fiber terminals in slices prepared from control and epileptic mice. Arrows mark the presence of silver-sulfide labelled mossy fiber terminals sprouted aberrantly into the inner molecular layer and on granule cell somata in epileptic tissue. Timm's staining repeated in 5 control and 12 epileptic slices. **b,** Proportional DGC activation (%) to 400 µA perforant path stimulation in control ACSF, 5 µM UBP-310, with before after plot of each slice on right. Control two-tailed, paired t-test t(8) = .3811, p = 0.7131. Epilepsy: one tailed, paired t-test, t(11) =2.619, p= 0.0119. Black plot is mean +/- S.E.M, gray are individual paired measurements. Sample sizes are as follows (slices): Control ACSF: n= 8; Glutamine: n = 8; UBP-310: n = 8; Epilepsy, ACSF: n= 12; Glutamine: n = 8; UBP-310: n = 12. Slices taken from at least 3 mice. Scale bars represent 50 µm.

Supplementary Videos S1-5 Confocal micrograph videos of OGB-1-loaded DGCs from control mice (**S1**), and mice 3-7 days (**S2**), 14 days (**S3**), 2-3 months (**S4**) and 6 months (**S5**) post-SE activating in response to 400 µA PP stimulation (1Hz, 10 pulses). Playback speed is in real time. White circle in bottom right indicates timing of PP stimulation. Note the markedly increased DGC activation compared to controls at 3-7 days, 2-3 months and > 6 months post-SE.
